# Opportunities and Challenges in Antibody–Drug Conjugates for Cancer Therapy: A New Era for Cancer Treatment

**DOI:** 10.3390/cancers17060958

**Published:** 2025-03-12

**Authors:** Idil Buyukgolcigezli, Ates Kutay Tenekeci, Ibrahim Halil Sahin

**Affiliations:** 1 Faculty of Medicine, Hacettepe University, Ankara 06230, Turkey; ibuyukgolcigezli@hacettepe.edu.tr; 2 Department of Biochemistry, Faculty of Medicine, Hacettepe University, Ankara 06230, Turkey; atesktenekeci@gmail.com; 3 Department of Pathology, University of Texas Southwestern Medical Center, Dallas, TX 75390, USA; 4 Division of Hematology/Oncology, University of Pittsburgh School of Medicine, Pittsburgh, PA 15213, USA

**Keywords:** antibody-dependent cellular cytotoxicity, antibody–drug conjugate, antibody therapy, cancer therapy, complement-dependent cytotoxicity, cytotoxic cancer therapy, DNA agent, topoisomerase-1 inhibitor, tubulin inhibitor

## Abstract

The antibody, linker, and payload moieties all play a significant role in giving the ADC its unique therapeutic potential. The antibody subclass employed in ADCs is determined based on relative individual receptor affinities and pharmacokinetics. Meanwhile, the linker used in an ADC can either be cleavable or non-cleavable. ADC therapy comprises antibody-dependent mechanisms in addition to the direct cytotoxic effects of the payload. These include antibody-dependent cellular cytotoxicity, complement-dependent cytotoxicity, and antibody-dependent cellular phagocytosis, as well as the “bystander effect”, which refers to the diffusion of a portion of the cytotoxic molecules out of the target cell, exerting its cytotoxic effect on the adjacent cells. Target antigens of ADCs are expected to be expressed on the membranes of the cancer cells facing the external matrix, although new approaches utilize antigens regarding tumor-associated cells, the tumor microenvironment, or the tumor vasculature. These target antigens of ADCs not only determine the efficacy of these agents but also impact the off-targets and related adverse effects. The majority of ADC-related toxicities are associated with off-targets. The proposed mechanisms of ADC resistance include disrupted intracellular drug trafficking, dysfunctional lysosomal processing, and the efflux of the cytotoxic molecule via ATP-binding cassette (ABC) transporters. The latter mechanism is especially prominent for multi-drug-resistant tumors. An important limitation of ADCs is the penetration of the conjugate into the tumor microenvironment and their delivery to target cancer cells. Cancerous tissues’ vascular profile and the steric “binding site barrier” formed around the peripheral vessels of tumors stand as potential challenges of ADC therapy for solid tumors. As research efforts focus on reducing toxicities, overcoming resistance, and improving pharmacokinetics, ADC options for cancer therapy are expected to continue to diversify, including standalone approaches and combination therapies.

## Simple Summary

Antibody–drug conjugates (ADCs) comprise a cytotoxic small molecule paired with a monoclonal antibody via a chemical linker. The antibody component reduces the cytotoxic effects on off-targets, making ADCs a popular area of research for the treatment of hematologic malignancies and solid tumors. This review article aims to provide an overview of ADCs in today’s cancer therapy landscape. We summarize the currently used ADCs with their cytotoxic payloads, targets, and therapeutic indications. We discuss the mechanisms of action of ADCs, as well as the associated toxicities and newly emerging mechanisms of drug resistance. We also elaborate on some of the current efforts to advance present ADC strategies, as well as opportunities for the future of ADC-based cancer therapy.

## 1. Introduction

In the early 1900s, William B. Coley, an orthopedic surgeon, administered bacterial mixtures, including *Streptococcus pyogenes* and *Serratia marcescens*, to his sarcoma patients with the goal of triggering a systemic immune response that would possibly help treat the tumor [1]. The administration of “Coley’s toxins” proved to be successful over the course of his career [2]. Roughly around the same period, German physician and pharmacologist Paul Ehrlich proposed a milestone concept, “magic bullet” drugs, which are directly targeted at diseased cells and do not impact others, named precision medicine in the modern era [3]. His work on immunological descriptions of antigens and receptors, as well as his work on targeting affinitive chemical substances to pathogens, led to him receiving the 1908 Nobel Prize for Physiology or Medicine [4]. Following Coley and Ehrlich, sequential efforts for the development of many cancer therapeutics took place in the 1900s, including DNA analogs, antimetabolites, cytotoxic molecules, protein tyrosine kinase inhibitors, and finally, monoclonal antibodies [3]. Coley’s vision of immunotherapy and Ehrlich’s targeted approach came to life when gemtuzumab ozogamicin was approved by the FDA for adults with AML in 2000, making it the first FDA-approved ADC [5,6].

An ADC is a therapeutic agent that is traditionally made from the chemical conjugation of a small molecule cytotoxic drug and a monoclonal antibody via a linker molecule [6]. The systemic administration of cytotoxic drugs leads to many adverse effects such as cytopenia, hair loss, and fatigue; hence, the targeted approach offered by ADCs is deemed to reduce off-target toxicity, enhance drug delivery, and improve therapeutic precision and drug efficacy [7]. Classic monoclonal antibody therapy can induce cellular killing by inducing antibody-dependent cellular cytotoxicity (ADCC) and/or interfering with signals that induce cellular growth, invasion, and migration [8]. However, monotherapy with mAbs for cancer treatment has not produced the desired deep and durable results [5]. ADCs have become prominent due to their dual-component nature, which potentially offers a fine balance between selectivity and toxicity, minimizing the effects on normal tissues by delivering the right therapy to the right target [9]. Overall, the toxicities associated with ADCs have been reported to be manageable, albeit varying depending on target and payload dose [10]. A prominent example regarding toxicity is DESTINY-CRC-01, a phase 2 trial investigating trastuzumab deruxtecan for HER2-expressing metastatic colorectal cancer. In this study, drug-induced ILD/pneumonitis was reported in 9.3% of patients, and 3.5% of patients had grade 5 ILD/pneumonitis, highlighting the potential significant toxicities of ADCs even with well-established targets [11].

This review discusses the mechanisms of action and the therapeutic advantages and disadvantages of cytotoxic ADCs, as well as an overview of novel ADC approaches currently in development. We also elaborate on the toxicities of these agents, resistance mechanisms, and the potential clinical use of ADCs for the future management of patients with cancers.

## 2. The Structure of an ADC

An ADC is typically made of a monoclonal antibody, a linker, and a cytotoxic small molecule. All three components, along with the method(s) by which they are conjugated, are critical in determining the biophysical and physiologic tendencies of the conjugate as a whole [12].

### 2.1. The Antibody Moiety and Target Antigen

The majority of FDA-approved antibody therapeutics, including ADCs, are of the IgG1 subclass [13], owing to the relatively long serum half-life [14] and enhanced effector functions [15]. It is also important to note how the weight of IgG averages at around 150 kDa, a nearly ideal value for target recognition and retention by cancer cells [16]. The IgG2 subclass is not commonly preferred for ADCs due to its relatively low affinity for the Fcγ receptor [17] and, perhaps more importantly, due to its tendency to form dimers and aggregates [18], an undesired condition for ADC development and design. The preference for IgG1 over IgG3 can be explained by IgG3’s short half-life due to an amino acid difference at position 435 [19], even though IgG3 has the strongest effector functions among all subgroups [17]. While it has a similar half-life to IgG1, IgG4 has a weaker affinity with phagocytic cells and can only induce limited immune response [17]. Hence, aside from instances where the immune response is deliberately toned down for safety concerns, IgG1 is the preferred subclass for ADC design [17].

One obvious function of the antibody moiety is the selective delivery of the payload to cancer cells that express the target antigens. Hence, the selection of the appropriate antigen is critical for the adequate functioning of the antibody. The target antigen should be surface expressed with exclusively higher levels on the cancer cells, and its expression on the healthy cells should be lower than that of cancer cells—if any at all [20]. The antigen should not be secreted into the circulation as that would amplify any possible undesired on- and off-target effects [6]. The binding of the antigen and the antibody should be of high affinity and selectivity. Ideally, the antigen should face the external matrix on the surface of the cancer cell and should be suitable for internalization as part of the antigen–antibody complex after the binding [21]. Finding a good antigen–antibody pair for ADC delivery is a challenge that requires careful considerations regarding the drug delivery as a whole, as it has been reported that antigen–antibody binding with very high affinity can also be detrimental to the drug delivery, possibly hindering thorough distribution of the drug into solid tumors [12]. Possible target antigen groups include surface proteins, glycoproteins, or abnormally expressed gangliosides [17]. The currently targeted antigens are proteins overexpressed on cancerous cells, and they include Trop2, nectin4, HER2, and certain CDs like 19, 22, and 33 [6]. A typical antigen example would be the HER2 receptor, which can be expressed at up to 100-fold levels in cancerous cells compared to healthy cells [6]. Although the conventional approach is to opt for surface-expressed cancer antigens like the ones mentioned previously, there are efforts to target tumor-associated cells [22] or antigens associated with the vasculature or stroma of the cancer microenvironment, such as TM4SF1 [23] or collagen IV [6]. ADCs that target the tumor matrix could have increased ways of damaging cancerous cells, as they can impact not only the cancerous cells, but also the interaction of the cancer cells with the supporting environment and surrounding cells of the immune system. The possibility of diversifying the target cells to genomically stable cells like those of the TMA can confer the ADC with an advantage over mechanisms of resistance that develop via mutations [6]. Engineering the targeting and effector pair requires great attention as the conjugation of the cytotoxic moieties to the antibody should not alter how the antibody circulates in the bloodstream nor reduce its affinity for the target antigen [5]. Following the delivery, the conjugate is to be internalized—the method of which is also largely determined by the target antigen [6]. The speed and efficacy of this internalization, on the other hand, depends on the epitope of the antibody and the conjugated payload, as well as the target antigen [17].

### 2.2. The Chemical Linker

**Figure 1:**
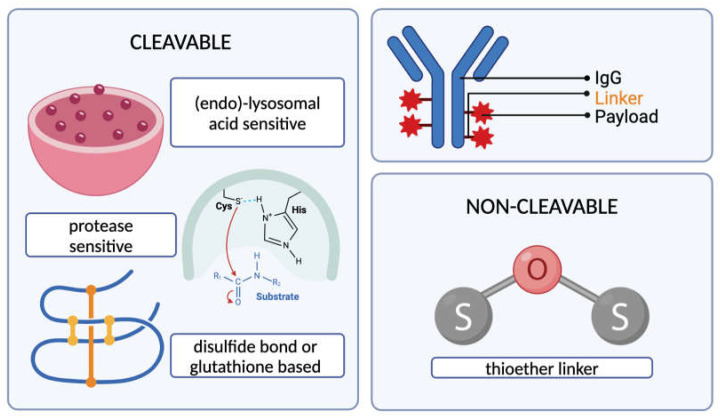
Types of cleavable and non-cleavable ADC linkers (created with BioRender.com, accessed on 15 February 2024).

The linker molecule connecting the antibody and the cytotoxic drug should keep the conjugate in bound form until the desired target site is reached [24] and is, hence, an important determinant of the pharmacokinetics and the therapeutic index of the conjugate [5]. Aside from circulatory stability and targeted release, one other key characteristic of a good linker is its low hydrophobicity, due to the fact that hydrophobic linkers can interact with the hydrophobic payloads, leading to aggregates that can possibly cause an immune reaction [25]. Linkers are principally categorized into two: cleavable and non-cleavable linkers. Cleavable linkers release the payload either upon interacting with the low pH environment of the (endo)-lysosome, e.g., hydrazone linkers [26], or upon direct cleavage via lysosomal cathepsins [27], e.g., peptide linkers. A subgroup of cleavable linkers contains disulfide bonds that are sensitive to intracellular glutathione, releasing the payload upon the chemical reduction in these bonds by glutathione [28]. Such linkers might prove useful for the development of future ADCs for many cancers, including ovarian, head and neck, and lung cancers, since increased tumor glutathione levels have been reported in the literature for these cancers [5,29]. Non-cleavable linkers generally present higher levels of plasma stability [26] but require the cytotoxic molecule to remain active after complete lysosomal degradation [27]. Although the latter might appear as a disadvantage at first sight, there are reports of enhanced ADC cytotoxicity when conjugated with a non-cleavable linker in comparison to cleavable ones [27]—as demonstrated by the development of Trastuzumab-MCC-DM1 (Trastuzumab emtansine) by Phillips et al. [30]. Site-specific degradation mechanisms of non-cleavable linkers are actively being researched to improve their efficacy over cleavable counterparts [31]. A summary of the types of linkers employed in ADC design is shown in Figure 1.

### 2.3. The Cytotoxic Payload

**Figure 2:**
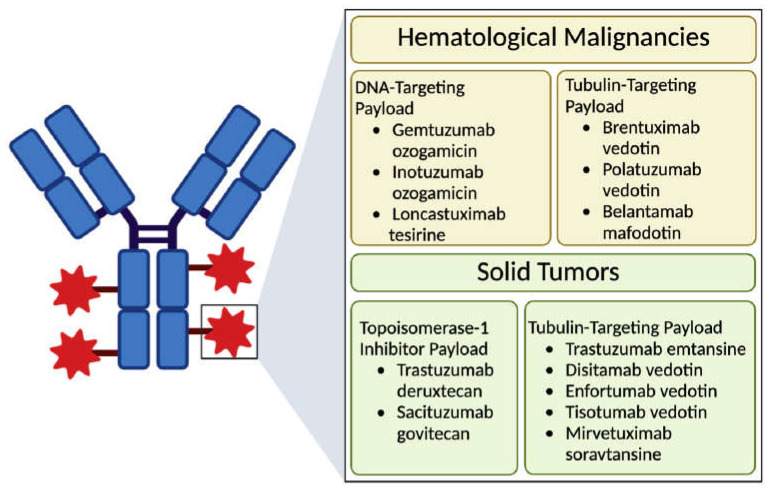
The categorization of current ADCs based on their payloads and targets (created with BioRender.com, accessed on 15 February 2024).

The majority of the payloads employed in currently active ADCs target DNA, disrupt microtubule organization [32], or inhibit topoisomerase 1 [6] (Figure 2). Despite the enhanced selectivity attained with the coupling of targeted antibodies, only 2% of IV-administered ADCs are successfully delivered to the tumor site [6]. Therefore, cytotoxic molecules that are nearly 1000 times more potent than standard chemotherapeutics are utilized for ADCs [33]. Consistently, warheads employed in ADCs exhibit high potency and show their IC_50_ even in a picomolar range [5,16].

The DNA-damaging agents used in ADCs prominently include calicheamicin, duocarmycin, and pyrrolobenzodiazepine dimers [16]. Calicheamicin binds to the minor groove of DNA, generates free radicals, and causes double-strand breaks [6]. Even though it is more commonly preferred as a payload for ADCs that target hematologic malignancies, such as gemtuzumab ozogamicin or inotuzumab ozogamicin [6], the recent literature provides evidence of ongoing research for its usage in solid tumors [34]. Duocarmycin’s mechanism of action is through the alkylation of adenines [6], and it has been popularly researched for new ADCs. Recent literature reports of comparative ADC studies show in vivo antitumor activity of a duocarmycin-based ADC for a murine SNU-16 model with no apparent toxicities [35]. Duocarmycin’s feasibility in conjugate therapies has been tested for various cancers. For instance, an anti-HER2 antibody–mimetic drug conjugate that utilizes duocarmycin has shown an effective reduction in tumor size in mice models [36]. An investigational ADC, vobramitamab duocarmazine, was shown to increase survival in both orthotopic and resected models of neuroblastoma (NB) and appears to be more favorable compared to the standard-of-care-therapy for relapsed NB [37]. Pyrrolobenzodiazepine is a potent molecule that creates crosslinks in the DNA [6]. A pyrrolobenzodiazepine-based ADC, ADCT-601, has been tested for various AXL-expressing cancers and exhibited antitumor activity in a monomethyl auristatin-E-resistant lung cancer model [38]. In a patient-derived xenograft model of pancreatic cancer, ADCT-601 was found to be more efficacious compared to a monomethyl auristatin-E-based ADC, and it resulted in complete tumor eradication following a single low-dose administration [38]. Pyrrolobenzodiazepine has also been investigated as a potentiator of some other cancer therapeutics, such as bortezomib and ibrutinib, for hematological malignancies [39]. One particular study was based upon the downregulatory effects of DC-1-192 (a pyrrolobenzodiazepine monomeric hybrid) on NF-кB since NF-кB overexpression was associated with resistance to chemotherapy in chronic lymphocytic leukemia (CLL) and multiple myeloma (MM) [39]. The combination of DC-1-192 with bortezomib and ibrutinib showed enhanced cytotoxic effects, and there was notable synergy between DC-1-192 and ibrutinib on a co-culture model of tissue-resident CLL cells [39]. It is important to note that ADCs conjugated with DNA-damaging agents are arguably deemed to be more effective than those conjugated with tubulin inhibitors, since DNA-damaging agents can show their actions irrespective of the stages of the cell cycle [6]. Further studies are needed to better define the comparative efficacy of DNA-damaging payloads in ADCs.

Cytotoxic molecules that target tubulin organization are primarily auristatins, maytansinoids [16], and tubulysins [6]. Auristatins constitute the majority of the currently employed payloads, and they exert their effect by disrupting microtubule polymerization, eventually leading to cell cycle arrest and cell death [24]. Prominent examples of auristatin-derived ADCs include Brentuximab vedotin and Polatuzumab vedotin [6]. The popularity of auristatins is mainly due to their chemical composition, which is hydrophilic, and they provide successful conjugation with the antibody moiety, improving the efficiency of the conjugate in vivo [40]. As demonstrated in trastuzumab emtansine [32], maytansinoids have their own binding site on the tubulin molecule [41], inhibiting tubulin polymerization [6]. Despite their exceptionally high potency of cell cycle arrest at the G2/M phase, maytansinoids’ complex chemical structure renders this payload subgroup relatively more difficult for drug development [41]. While being clinically investigated to a much lesser extent, tubulysins constitute another subgroup that inhibits tubulin polymerization [6]. Research regarding the conjugation of tubulysins is particularly important as tubulysins cannot be effluxed out of the cell via P-glycoprotein pumps that efflux maytansinoids and are hence proposed to be effective against multi-drug-resistant tumors [42]. There are ongoing efforts to limit tubulysins’ extreme toxicity and possibly utilize this payload for treating MDR1 tumors [43]. An example of tubulin-derived ADCs is the third-generation conjugate MEDI4276, which targets HER2 [32]. While these agents function by inhibiting tubulin polymerization, there are also some other molecules that disrupt the cell cycle by enhancing tubulin polymerization, e.g., discodermolide [44] and docetaxel [45], that are actively being investigated for use in ADCs [41]. Although the conjugation of docetaxel with panitumumab has been reported to result in a decrease in tumor size and overall survival in A431 tumor-bearing nude mice, this experimental ADC was concluded to be less efficacious than the combination of panitumumab and docetaxel therapy [45].

Topoisomerase-1 inhibitors are a more recent group of cytotoxic agents and have been successfully used in Trastuzumab deruxtecan (T-DXd) and sacituzumab govitecan [24]. Deruxtecan is the potent topoisomerase inhibitor found in the structure of T-DXd, and it creates a bystander-killing effect on the surrounding cells, regardless of their HER2 expression [46]. T-DXd was tested on patients with HER2-low metastatic breast cancer and was found to show a 50% lower risk for disease progression or death than the physician’s choice chemotherapy, irrespective of the expression of HER2 [47]. The bystander-killing effect on the surrounding cells explains the success of this targeted therapeutic despite low expression of the target. Recently, the DESTINY-Breast03 data showed improved outcomes with Trastuzumab deruxtecan (Enhertu) compared to Trastuzumab emtansine (T-DM1), with a longer duration of progression-free survival among patients with HER2-positive breast cancers (28.8 months vs. 6.8 months) [48]. T-DXd has been approved by the FDA as the standard second-line therapy for HER2-positive breast cancer [24]. It has also been approved for HER2-positive gastric cancers [49], and recent studies indicate it has activity in other solid tumors with HER2 amplification, such as colorectal cancer [11]. Sacituzumab govitecan (SG), on the other hand, targets possible overamplifications of Trop-2 in solid tumors, which notably include triple-negative breast cancer [6]. Like T-DXd, SG also exhibits bystander killing [50]. SG’s linker CL2A arguably contributes to the targeting of the conjugate as it connects the payload to the antibody with a higher affinity in the tumor sites when compared to non-target sites [6]. This renders the conjugate with a longer half-life in target tissues when compared to non-target tissues [6], presenting a possibly more advantageous strategy for controlled toxicities. A randomized phase III study comparing SG with physician’s choice for the treatment of previously treated metastatic triple-negative breast cancer (LBA17 ASCENT) was conducted during the acceleration of the FDA approval. It was shown that SG provided a significantly improved median progression-free survival (5.6 months vs. 1.7 months) as well as a better objective response rate (35% vs. 5%) in comparison to the physician’s choice [50]. One other particularly interesting expansion brought forward with the aforementioned two ADCs employing topoisomerase-1 inhibitors is how both conjugates were manufactured with relatively high DAR values of around 8 [24]. This is remarkable as it underscores the possibility of retaining the pharmacokinetics and solubility profile of a conjugate, despite the addition of more cytotoxic moieties [24]. Theory building on these examples could possibly diversify the ADC scene as it can suggest that there might be room for the employment payloads that are traditionally deemed too weak to be made part of ADCs, given that they are conjugated at high enough DARs [24].

**Figure 3:**
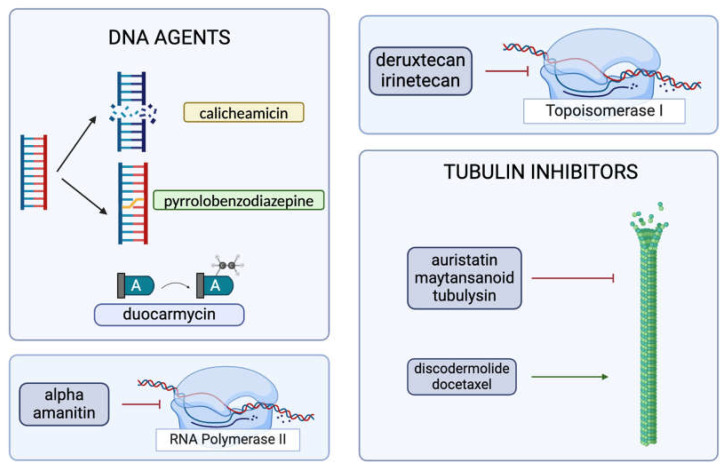
The classification and action mechanisms of currently employed ADC warheads (created with BioRender.com, accessed on 15 February 2024).

A relatively new payload that is being investigated is an RNA polymerase II inhibitor called alpha-amanitin; it inhibits DNA transcription [24] and hence presents increased potency as it affects both proliferating and non-proliferating cells [51]. While promising, it is important to note that alpha-amanitin is quite new; tubulin inhibitors and DNA-damaging agents continue to evolve in the field rapidly (Figure 3). The currently approved ADCs are summarized in Table 1, along with their payload categorization and their approval status.

## 3. Mechanisms of Action of ADCs

The interaction of ADCs with the target antigen often forms antigen–antibody binding, which then triggers the internalization of the ADC–antigen complex [24]. While the internalization most commonly occurs via clathrin-mediated endocytosis, there are ongoing efforts to engineer the antibodies to exploit the endolysosomal systems [52]. It is also important to note that clathrin-independent mechanisms have also been proposed for the internalization of ADCs, which include caveolae-mediated endocytosis, CLIC-GEEC endocytosis, and macropinocytosis [53]. The stages of ADC internalization and the intracellular trafficking of the ADC complex are among the understudied aspects of ADC metabolism and require further research.

**Figure 4:**
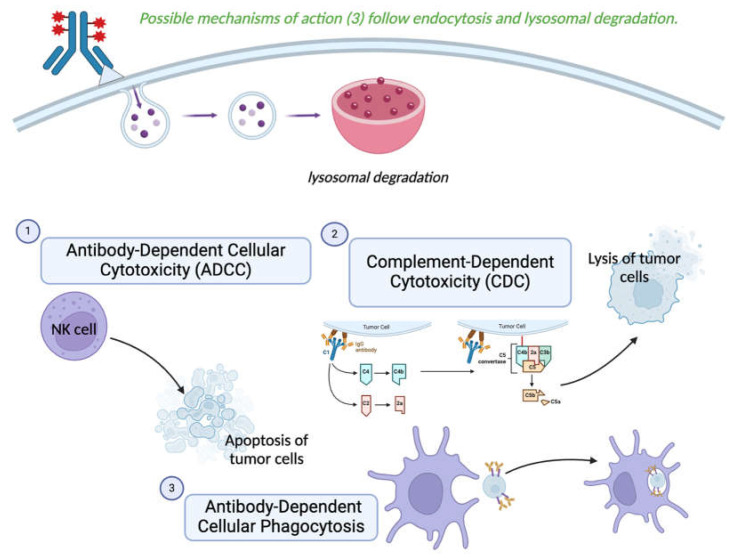
Cellular killing mechanisms mediated by the antibody component of an ADC (created with BioRender.com, using complement activation pathway grouped icon, accessed on 15 February 2024).

Upon internalization and lysosomal degradation, the cytotoxic molecule is released and shows its effect via the numerous possible mechanisms discussed above. It is important to note that a portion of the cytotoxic molecules can then diffuse out of the cell, promoting an indirect cytotoxic effect on the nearby cells [24]. This phenomenon is known as the “bystander effect” and is an area for further research [54]. In addition to the cytotoxic effects, the antibody moiety of an ADC can also induce significant antitumor effector functions. These include complement-dependent cytotoxicity (CDC), antibody-dependent cellular cytotoxicity (ADCC), and antibody-dependent cellular phagocytosis, and they differ from the mechanisms associated with conventional immunotherapy [6]. Although enhancing the ADCC activity of ADC appears to be promising [15], it is important to note that ADCC is difficult to test in murine models due to its species-specific nature [55]. Suggested mechanisms for improving ADCC and CDC include amino acid modifications in the Fc fragment or mixing the constant regions of different antibody subclasses, such as IgG3 and IgG1 [15]. Such antibody-dependent effector functions may enhance the overall impact of the conjugate (Figure 4).

## 4. Limitations of an ADC

Early ADCs were targeted toward hematological malignancies, mainly due to challenges in the delivery of ADCs to a solid tumor site, as only 2% of the administered ADCs make it to the tumor site [6]. Hence, the progress of drug development with ADCs for solid tumors has evolved at a slower pace. The penetration of the drug into the solid tumor microenvironment presents a challenge for drug development and also creates challenges due to toxicity related to off-target effects, as seen in the ADCs that have been discontinued [24].

The penetration of any drug into a solid tumor depends mostly on the effusion of the molecule through the characteristically permeable vessels of the tumor—a phenomenon known as the “Enhanced Permeability and Retention Effect” [56]. The vessels of a tumor usually have wide lumens, numerous irregular branches and shunts, a reduced number of surrounding pericytes, and many fenestrations [57]. This abnormal nature of the tumor vasculature normally allows treatment with larger molecules such as conjugates; however, the tumor microenvironment can also complicate ADC treatment due to obstruction of the lymphatic or blood vessels of the tumor or elevation of the intravascular hydrostatic pressure [58]. Similarly, the aggregation of the antibody–antigen complexes around the vascularized areas of the tumor can sterically hinder and prevent the further attachment of antibodies, creating a “binding site barrier” [59]. This effect is also observed in standard antibody therapies, but it results in a much more significant reduction in the efficacy of ADC therapeutics, as ADCs are administered at relatively lower doses due to their cytotoxic components [60].

### 4.1. Toxicities

The toxicity of ADCs remains an important challenge despite the fact that the antibody moiety enhances the selectivity of cytotoxic agent delivery over standard chemotherapeutics. The off-target effects concern the binding of the antibody moiety to antigens other than the target antigen, while the on-target but undesired effects refer to the binding of the antibody to the target antigen expressed in healthy tissues [24]. The target itself is an important determinant of the adverse effects and toxicities since some antigens are commonly expressed on otherwise healthy noncancerous cells. Linker instability is also important as the premature release of the cytotoxic small molecule into circulation can result in widespread off-target toxic effects [32], given that some cytotoxic payloads can be carried in plasma bound to albumin to healthy tissues [24]. ADC toxicity most commonly presents with severe hematologic toxicities such as thrombocytopenia, neutropenia, and anemia [6], along with ocular toxicity, which is most likely due to the rich blood supply and the receptor expression profile of the eye [61]. ADC-related ocular toxicities can exhibit corneal deposits, keratitis as well as decreased visual acuity, as seen in the trial of the ADC Cantuzumab ravtansine for Can-ag-expressing tumors [16]. Ample vascularization and the presence of certain mannose receptors in hepatic cells make the liver another off-target for toxicities [24]. For example, Trastuzumab emtansine has been reported to be linked to increased AST (4.3%) and increased ALT (2.9%) levels [6]. Another notable example of the adverse effects of current ADCs is the occurrence of T-DXd-induced ILD in both HER2-expressing metastatic colorectal cancer [11] and non-small-cell lung cancer (NSCLC) [6]. DESTINY-Lung01 data showed that ILD was observed in 26% of the NSCLC patients [6], while DESTINY-CRC01 data showed drug-induced ILD in 9.3% of the patients [11]. It appears that dose adjustment can alleviate the grade of ADC toxicities. For instance, 5.4 mg/kg T-DXd dosing showed a better safety profile compared to 6.2 mg/kg for patients with HER2-amplified mCRC treatment with no grade 5 toxicity in DESTINY-CRC02, while treatment-related mortality was noted in DESTINY-CRC01 [62]. Notably, unexpected toxicities are among the main reasons for the termination of an ADC’s pipeline. Such examples include HTK288 (ADC against cadherin-6), leading to CNS toxicity, and LOP628 (ADC against KIT), leading to severe hypersensitivity reactions [24].

Collectively, growing evidence indicates that ADCs can induce significant toxicities due to their payloads, and the precision approach with these agents does not preclude significant toxicities, which should be considered when developing novel ADCs in the future. Perhaps the most dispositional way to go about reducing ADC toxicities would be to opt for conjugates with less cytotoxic payloads in the first place. Other approaches would include bettering the site-specific activation of the cytotoxic moieties through improved engineering of linkers and/or masked domains, as well as the discovery of target antigens that are exclusive to the tumor or its microenvironment.

### 4.2. Mechanism of Resistance

**Figure 5:**
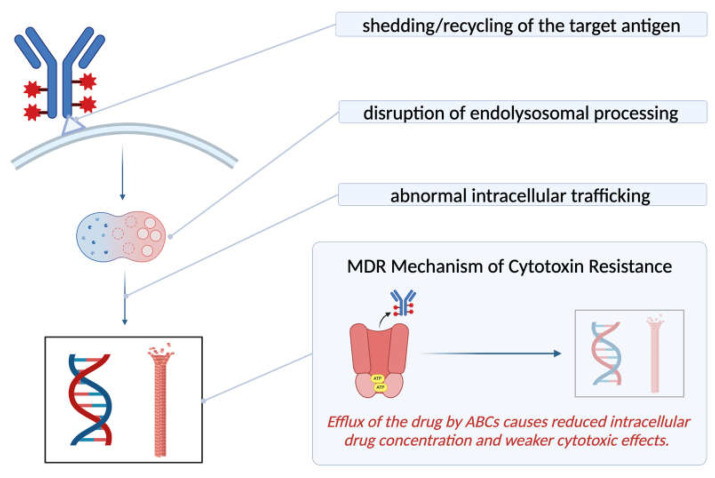
Steps of ADC delivery and action at which therapeutic resistance can possibly emerge (created with BioRender.com, accessed on 9 March 2025).

Drug resistance is one of the emerging challenges of ADCs, partially due to cancer cells’ tendency to develop a gradual resistance toward any treatment with mAb components [5]. Three main mechanisms have been proposed for ADC resistance, including the disruption of intracellular drug trafficking, abnormal lysosomal function, and resistance associated with the cytotoxic molecule itself [63]. The latter mechanism is especially important for the development of MDR (multi-drug resistance) [5], which stands as one of the most significant clinical challenges of chemotherapy [64]. Although MDR can occur via alterations in the cancer niche or via the firing of anti-apoptotic pathways [64], a particularly significant sub-mechanism is the removal of the cytotoxic agents via efflux pumps such as ATP-binding cassette (ABC) transporters [6], leading to reduced intracellular drug concentration [63]. Other mechanisms of resistance include the deliberate reduction in the expression of target antigen on the cancer cell [6] and the rapid recycling of the antigen–antibody complex to the cell membrane [5]. The latter results in the evasion of ADC lysosomal degradation, which is necessary for its therapeutic action. The efficiency of an ADC depends on multiple steps that include the successful delivery of the ADC complex to the tumor microenvironment, high-affinity binding of the antibody to the target cell surface antigen, and the rate at which it is successfully internalized and processed in the cancer cell. Hence, any barrier that may impact the drug delivery and internalization process can manifest as one of the limitations of ADCs and result in therapeutic resistance (Figure 5) [26].

## 5. Discussion and Future Perspective

Regarded as “magic bullets”, ADCs have started to revolutionize oncology research with their dual nature, resulting in many success stories. An example is the auristatin-derived ADC Brentuximab vedotin and its sequential approval for Hodgkin’s lymphoma, systemic anaplastic large cell lymphoma, primary cutaneous anaplastic large cell lymphoma, and certain types of peripheral T-cell lymphoma [6]. Research for novel ADC payloads is also promising as Moxetumomab pasudotox—an immunotoxin-derived ADC—became the first new drug approved for hairy cell leukemia treatment in over a decade, marking a breakthrough [6].

Most ongoing research for the optimization of ADCs entails improving the linker chemistry, reducing the off-target effects of the conjugate, or enhancing the internalization of the target receptors. It is important to note that the majority of the cytotoxic molecules employed in an ADC are still either tubulin or DNA targeting and that a portion of the current research focuses on the diversification of the cytotoxic payloads with different mechanisms of action. Such efforts include studies with antimitotic kinesin spindle protein inhibitors, thalianstatins that target the spliceosome, nicotinamide phosphoribosyl transferase inhibitors (NAMPTs), and peptidic proteasome inhibitors like carmaphycins [28]. With the combination of payloads that have different mechanisms, heterogenous tumors can be targeted more effectively. For example, in the study by Yamazaki et al., a dual payload resulted in promising responses in a T-DM1-resistant HER2-expressing breast cancer model [65].

Optimization of the site-specific activation of the conjugate can play an important role in reducing systemic effects and has been an objective of recent research. Kang et al. reduced the affinity of a HER2-specific antibody to its receptor in acidic pH, engineering another “cleavage” step and hence improving the pharmacokinetics of the conjugate [53]. Such modifications regarding activation in a low pH range can possibly facilitate more specific targeting of tumors since the cancer microenvironment is more acidic. The recent literature reports experimentation with several new target antigens, including RAGE (Receptor for Advanced Glycation End Products) for endometrial cancer [66] and ICAM1 (Intracellular Adhesion Molecule 1) for triple-negative breast tumors [67]. In addition to seeking more specific cancer cell surface antigens, several other mechanisms have been investigated for the prevention of ADC-associated on-target undesired effects. These include the administration of the antibody as an initially inactive “probody” that only gets activated in the cancer niche [68] or the usage of engineered bispecific antibodies with an initially masked binding domain [69]. The latter approach was investigated for conditional B cell lymphoma targeting in 2023 by Schoenfeld et al. and showed the value of utilizing the matrix metalloproteins of the cancer microenvironment [70]. Some of the most recent research for better targeting the ADCs to the tumor site includes using an antibody that has anti-payload fragments, helping attract more of the warhead molecules to the tumor site. This “inverse-targeting” approach brings any freely circulating cytotoxic molecule into the vicinity of the target cell and keeps it bound to the ADC until internalization, presenting a better safety profile. Payload-binding mAbs in ADCs have been successfully tested by Nguyen et al. for a maytansinoid derivative anti-CD123 ADC, exhibiting improved efficacy [71]. Bordeau et al. tested payload-binding mAbs for auristatin derivative ADCs, including Polotuzumab vedotin and trastuzumab-vc-MMAE, and they concluded that this method allowed for a potentially better therapeutic window since it presents decreased off-target toxicity and unchanged antitumor efficiency [72]. The decreased off-target toxicity is due to how the anti-payload mAb fragments bind and neutralize the freely circulating monomethyl auristatins in plasma, allowing for the renal clearance of the neutralized compound [72].

One proposed method for improving ADC efficacy has been combining ADC treatment with other targeted approaches, like mAbs. In the past, the co-administration of antibodies and antibody-based therapeutics have been reported to be successful [73]. However, in a study using N87 and MDA-MB-453 xenograft models, Singh et al. showed that the success of co-administrating Trastuzumab-based ADCs with Trastuzumab depends on the antigen expression profiles and that the benefit obtained cannot be generalized for all tumors [60]. Similarly, Menezes et al. co-administered a TAK-164 (an ADC) with 5F9 mAb (an anti-GCC antibody) to primary human tumor xenograft (PHTX-11C) model mice. Although the results showed better penetration of the conjugate into the tumor, there was no significant improvement in overall efficacy due to reduced cytotoxic effects [74]. Ultimately, research regarding the combination of ADC with other mAbs continues to evolve, albeit so far showing limited applicability due to tumor and antigen specificity. Therapies that combine ADCs with targeted approaches go beyond mAbs and include inhibitors for HER2, Pi3K, tyrosine kinases, CDK4/6, Akt, and PARP [75]. Additionally, there are approaches combining ADCs with true immunotherapy, such as immune checkpoint inhibitors or co-stimulatory T-cell receptor agonists [75].

Although drug resistance is an obstacle to ADC development, evidence from preclinical models of treatment resistance against various conjugates remains limited [32]. Yamazaki et al. showed that working with a dual-payload ADC may at least partially address intratumor clonal heterogeneity-related drug resistance [65]. Other reported efforts to combat ADC resistance include overcoming the binding site barrier; Bordeau et al. investigated a deliberate temporary inhibition of the antibody–antigen binding [59]. In this study, co-administering a Trastuzumab inhibitor with Ado-Trastuzumab emtansine (T-DM1) to xenograft models of NCI-N87 mice enhanced efficacy and median survival [59]. The proposed mechanism is that the transient inhibition of the antigen–antibody binding allows time for better distribution of the conjugate within the tissue and prevents accumulation of the conjugate around the vascularized areas of the tumor. This hypothesis is supported by evidence derived from the co-administration of the inhibitor and Trastuzumab to SKOV-3 xenograft mice, which showed an enlarged area of the tumor that stained positive for the antibody [59]. This indicates better penetration of the antibody away from the vasculature and into the tumor under the presence of the inhibitor. In the same study, it was shown that the co-administration of the antibody with the T-DM1 resulted in increased T-DM1 efficacy and median survival in NCI-N87 xenograft mice [59]. The transient inhibition approach is interesting as it is independent of the protease expression of the tumor, providing a possible advantage over using probodies or masked antibodies [59]. Other proposed solutions to ADC resistance include but are not limited to the improvement of non-cleavable linkers, manipulation of the endolysosomal system, and perhaps more dispositionally, selection of payloads that are poor substrates of MDR transporters and efflux pumps [5].

It is important to note that although ADCs are typically considered to include a cytotoxic conjugate attached to the antibody as a payload, alternative conjugate formats are actively emerging. Radioconjugates, preferably for use in imaging and monitoring of cancers [76], and antibody-functionalized nanoparticles that can offer the direct delivery of liposomes [77] are in development. Another emerging group of possible payloads includes immunomodulatory agents, aiming for the conjugation of a cytokine in place of a cytotoxic molecule to increase tumor immunogenicity and promote antitumor immunity [78]. Degrader–antibody complexes, a new class of ADCs, are being researched for the degradation of protein targets via the PROTAC payload [79]. Aside from site-specific activation via these degrader complexes, the usage of antibody fragments instead of full-sized antibodies is also part of the current discussion. Conjugating the payloads to smaller targeting moieties that retain the VH and VL regions can offer several advantages, including better penetration into solid tumors, less cross-reactivity, and, hence, better tolerance [17].

The current popularity of ADCs in the oncology field is due to the future potential of ADC-based therapies that may exceed the predefined paradigms of targeted therapies. It is important to note that several attempts have been made to detect therapeutic cell surface markers, which will likely contribute to the development of novel ADCs for the management of patients with various cancers. Understanding the pharmacokinetics and pharmacodynamics of monoclonal antibodies and the barriers that play a key role in the delivery of ADCs to cancer cells, including the tumor microenvironment, will enable further optimization of ADCs for their clinical use. In fact, more precise delivery with a better-defined target will enhance the efficacy of ADCs while reducing the off- and on-target toxicities.

Of the several limitations of ADC therapy discussed previously, the one with the most pronounced clinical effects is still the issue of associated toxicities. One solution would be to research linkers with better stability, eliminating premature drug release into circulation. Probodies and engineered antibodies with initially masked domains can be of help, but in vitro trials of antibody therapies are challenging due to the species-specific nature of many of the antibody-dependent mechanisms. Therefore, further research can aim to improve the site-specific activation of the cytotoxic molecules. Since cancer research is becoming more centered around the tumor microenvironment, the acidic environment produced by the cancer cells’ hypoxia and lactate metabolism can be noted as possible targets for the aforementioned site-specific activation [80]. Cancer-associated enzymes like matrix metalloproteinases, hyaluronidase, and cathepsins are candidates for further research as possible facilitators of the site-specific drug delivery of ADCs [81]. ADC-based therapies are currently expanded with immune-stimulatory conjugates, the main aim of which is to amplify the immune response to cancerous cells. Whether with standard ADCs or the newly emerging immunoconjugates, the most dispositional way to avoid ADCs’ off-target effects will require more translational and clinical research. This idea has long been on the agenda for improving mAb treatments, and some of the most recent research focuses on utilizing the cancer microenvironment and tumor-associated cells as possible targets. This concept can be utilized to modify the immune-suppressive tumor microenvironment with an aim to optimize and enhance the response to immune checkpoint inhibitors and other immunotherapeutic agents. In fact, the tumor microenvironment of liver metastasis of solid tumors remains a major challenge for immunotherapy, where significant resistance is seen in various tumors [82,83]. While the research for better antigen identification continues to be an unmet need, the antigen expression profiles of tissues commonly involved in ADC-related toxicities can be used as valuable tools for predicting the adverse effects of a new ADC. This can, in turn, contribute to the drug optimization process with improved safety profile and enhanced efficacy.

One particularly understudied aspect of ADCs is their cellular metabolism; little is known about the endocytosis and further processing of the conjugate. This could be promising as manipulation of the endolysosomal pathways can both contribute to the solution of ADC resistance and be important for the bettering of the bystander effect. Studies aiming to better understand what triggers the endocytosis of antibody therapeutics can also bring opportunities for pharmacokinetic improvements. For example, a study conducted on ERBB2/HER2-specific antibodies has shown that the internalization happened via an aggregation-dependent endocytosis process that was largely dependent on the cross-linking of the extracellular domains of the antibodies—rather than the antigenic targets themselves [84]. This is a valuable piece of information that could possibly bring attention to better selection of antibodies based on their extracellular domains and/or supplementing them with secondary antibodies that might speed up the internalization. One study showed that using tetravalent antibodies instead of bivalent antibodies, along with FGFR1 receptor clustering, could induce fast and efficient uptake that can occur via both clathrin-dependent and dynamin-mediated clathrin-independent mechanisms [85]. However, it is important to consider the effects of antibody targeting and receptor clustering as means of endolysosomal manipulation in the context of ADCs, since the conjugate as a whole might behave differently than its components. The literature reports that the establishment of a protein scaffold via multivalent antibodies confers protein drug conjugates with increased methods of internalization, faster lysosomal processing, and less recycling [86]. If successfully extended to restructure the clinically approved ADCs, the concentration of the antibody moieties in tumor sites via these mechanisms could also potentially improve the bystander effect. An enhanced effect of one dose of the conjugate on the nearby cells can help lower the doses at which ADCs are administered, improving adverse events and patients’ quality of life. Another way to lower the dose needed for effective ADC treatment would be to resolve the binding site barrier formed around the vascularized periphery of solid tumors. This would require precise engineering of the mAbs and their ionic charges, as well as the manipulation of the turnover times of the receptors back to the cell membrane surface. The timeline of the recycling of the receptors to the cell surface is critical for fine-tuning the pharmacokinetics of the conjugates, highlighting the need for further analysis of ADCs’ cellular metabolism.

Collectively, ADCs are a highly promising class of drug with rapidly evolving research on the optimization of the conjugates’ efficacy and safety profile, comprising both ADC monotherapies and combination regimens with other cancer therapeutics, particularly with immunotherapy. In the next decade, we can expect to see the approval of more ADCs and the expansion of the therapeutic indications of some of the currently approved ADCs. Table 2 shows some of the ADCs that are currently in late-stage clinical trials.

## 6. Conclusions

Chemotherapy and mAb-based targeted therapies have both come a long way since the initial trials, and ADCs mark an important milestone in cancer treatment as a fine-tuned combination of the two. Although there are current efforts to enhance the pharmacokinetics and pharmacodynamics of ADCs and to diversify their substituents, it is important to realize the slow shift to alternative conjugate formats in the research field, which may open pathways for novel therapeutics with more durable efficacy. Immune-stimulating and immunomodulatory compounds have made their way into preclinical trials, and smarter molecules that can exploit novel cellular pathways are in the clinical pipeline. There is no doubt that ADCs have revolutionized the landscape of cancer management with their dual nature and that the selectivity offered by the antibody moiety is invaluable. Drug resistance and the challenges of drug penetration into solid tumors are among the many areas for improvement currently explored as the interest of researchers in bettering ADCs persists. We can expect to see more ADCs—and possibly immunoconjugates—in both monotherapy and combined regimens in the near future.

## Figures and Tables

**Table 1:** Examples of ADCs and their approval status in the USA * [6,24].

ADC	Commercial Name	Warhead + [Linker Type]	Target	Status	Indication
Gemtuzumab ozogamicin	Mylotarg^®^	Calicheamicin [cleavable]	CD33	Reapproved in 2017; initially in 2000	CD33+ AML
Brentuximab vedotin	Adcetris^®^	MMAE [cleavable]	CD30	Approved in 2011	R/R CD30+ HL and systemic ALCL
Inotuzumab ozogamicin	Besponsa^®^	Calicheamicin [cleavable]	CD22	Approved in 2011	R/R B-cell precursor ALL
Moxetumomab pasudotox	Lumoxiti^®^	PE38 (immunotoxin) [cleavable]	CD22	Approved in 2018	R/R HCL
Polatuzumab vedotin	Polivy^®^	MMAE [cleavable]	CD79B	Approved in 2019	R/R DLBCL
Belantamab mafodotin	Blenrep^®^	MMAF [non-cleavable]	BCMA	Approved in 2020	R/R MM
Loncastuximab tesirine	Zynlonta^®^	PBD [cleavable]	CD19	Approved in 2021	R/R Large B-Cell Lymphoma, DLBCL
Trastuzumab emtansine	Kadcyla^®^	DM1 (maytansinoid) [non-cleavable]	HER2	Approved in 2013	HER2+ Early or Metastatic Breast Cancer
Enfortumab vedotine	Padcev^®^	MMAE [cleavable]	Nectin-4	Approved in 2019	Metastatic Urothelial Cancer
Trastuzumab deruxtecan	Enhertu^®^	Deruxtecan (topoisomerase-1 inhibitor) [cleavable]	HER2	Approved in 2019	HER2+, HER2-low Breast Cancer, NSCLC, GC/GEJ Adenocarcinoma
Sacituzumab govitecan	Trodelvy^®^	SN-38 (topoisomerase-1 inhibitor) [cleavable]	Trop-2	Approved in 2020	TNBC, Metastatic Urothelial Cancer
Disitamab vedotin	Aidixi^®^	MMAE [cleavable]	HER2	Approved in 2021	Gastric Cancer
Tisotumab vedotin	Tivdak^®^	MMAE [cleavable]	TF	Approved in 2021	Cervical Cancer

* The first seven rows have been shaded to represent the ADCs targeted for hematologic malignancies. The remaining conjugates target solid tumors. Abbreviations: ALCL, anaplastic large-cell lymphoma; ALL, acute lymphoblastic leukemia; AML, acute myeloid leukemia; DLBCL, diffuse large B-cell lymphoma; GC, gastric cancer; GEJ, gastro-esophageal junction cancer; HL, Hodgkin Lymphoma; HCL, hairy cell leukemia; MM, multiple myeloma; MMAE, monomethyl auristatin E; MMAF, monomethyl auristatin F; NSCLC, non-small-cell lung cancer; PBD, pyrrolobenzodiazepine; R/R, refractory or relapsed; TNBC, triple-negative breast cancer.

**Table 2:** Selected ADCs in late-stage clinical trials [24].

Conjugate	Warhead	Target	Status	Patient Population
XMT-1536	Auristatin F-hydroxypropylamide	NaPi2b	Phase III	Ovarian cancer
SHR-A1811	Rezetecan	HER2	Phase III	HER2+ Breast cancer
ARX788	Amberstatin 269	HER2	Phase III	HER2+ Breast cancer
ABBV-399	Monomethyl auristatin E	MET	Phase III	Non-small-cell lung cancer
U3-1402	DXD	HER-3	Phase III	Non-small-cell lung cancer
SAR408701	DM4	CEACAM5	Phase III	Non-small-cell lung cancer
DS-1062	DXD	TROP2	Phase III	Breast cancer
SKB264	Belotecan	TROP2	Phase III	Triple-negative breast cancer
MK-2140	Monomethyl auristatin E	ROR1	Phase II/III	Diffuse large cell B-lymphoma
MGC018	Duocarmycin	B7-H3	Phase II/III	Prostate cancer
ADCT-301	PBD SG3199	CD25	Phase II	Hodgkin’s lymphoma, acute myeloid leukemia
IMGN632	DGN549 IGN	CD123	Phase II	Blastic plasmacytoid dendritic cell neoplasm
CX-2009	DM4	ALCAM	Phase II	Breast cancer
DS-7300a	DXD	B7-H3	Phase II	Small cell lung cancer
SGN-LIV1A	Monomethyl auristatin E	LIV-1	Phase II	Lung cancer
BA3011	Monomethyl auristatin E	AXL receptor tyrosine kinase	Phase II	Ovarian cancer, Non-small-cell lung cancer
BA3021	Monomethyl auristatin E	ROR2	Phase II	Head and neck squamous cell carcinoma, Non-small-cell lung cancer, ovarian cancer
MRG003	Monomethyl auristatin E	EGFR	Phase II	Nasopharyngeal carcinoma, Biliary tract cancer, Non-small-cell lung cancer, Head and neck squamous cell carcinoma
MRG002	Monomethyl auristatin E	HER2	Phase II	Breast cancer, Non-small-cell lung cancer, Urothelium cancer, Biliary tract cancer
DX126-262	Tub114 (Tubulysin B analogue)	HER2	Phase II	HER2+ breast cancer
MORAb-202	Eribulin	FR-α	Phase II	Non-small-cell lung cancer, Ovarian cancer

## Data Availability

Not applicable.

## Abbreviations

The following abbreviations are used in this manuscript:

MDPI	Multidisciplinary Digital Publishing Institute
DOAJ	Directory of open access journals
TLA	Three-letter acronym
LD	Linear dichroism
